# MIGC 2019 SYMPOSIUM: STRENGTH IN CREATING DIVERSIFIED SPACES IN EDUCATION AND RESEARCH ON AGING

**DOI:** 10.1093/geroni/igz038.1313

**Published:** 2019-11-08

**Authors:** Adrienne T Aiken Morgan, Candace Brown, Gregory R Samanez-Larkin

**Affiliations:** 1 North Carolina A&T State University, Greensboro, North Carolina, United States; 2 Duke University, Durham, North Carolina, United States

## Abstract

Populations of minority older adults will continue to increase at an accelerated pace in the coming decades. As such, it is increasingly important to disseminate minority aging education and research topics in spaces that will prepare gerontology scholars to address the needs of diverse elders. This symposium will highlight efforts to diversify academic spaces by scholars engaged in minority aging education and research. The first presentation describes a service-learning pedagogical approach to teaching minority aging topics to graduate students. It will discuss how a gerontological social work course seeks to offer real-world learning experiences through community partnership. The second presentation discusses an experiential learning pedagogical approach to teaching social determinants of health to graduate students at a historically Black university and highlights how to apply theoretical concepts to creating community needs assessments and health promotion programming for the local community. The third presentation discusses efforts to teach undergraduate students about older LGBT individuals, who represent a growing group of minority elders. This presentation advocates for the use of various strategies for integrating both research and pedagogical approaches to increase knowledge and awareness of LGBT aging topics. The last presentation focuses on the promotion and dissemination of scholarship produced at minority-serving institutions (MSI) through the creation of a new open-source journal. This presentation describes publication challenges for tenure-track MSI faculty and developed opportunities for inclusiveness of such scholarship. The symposium discussant will summarize these challenges, opportunities, and implications to promote minority-focused gerontological topics in academia.

